# Phase I/II clinical trial on the safety and preliminary efficacy of donor-derived anti-leukemia cytotoxic T lymphocytes for the prevention of leukemia relapse in children given haploidentical hematopoietic stem cell transplantation: study rational and design

**DOI:** 10.3389/fimmu.2025.1601961

**Published:** 2025-06-06

**Authors:** Daniela Montagna, Patrizia Comoli, Matteo Tanzi, Enrica Montini, Antonia Moretta, Gloria Taurino, Stella Boghen, Arianna Panigari, Tommaso Mina, Giovanna Giorgiani, Claudia Del Fante, Cesare Perotti, Marco Zecca

**Affiliations:** ^1^ Cell Factory, Fondazione IRCCS Policlinico San Matteo, Pavia, Italy; ^2^ Pediatric Clinic, Fondazione IRCCS Policlinico San Matteo, Pavia, Italy; ^3^ Department of Sciences Clinic-Surgical, Diagnostic and Pediatric, University of Pavia, Pavia, Italy; ^4^ Pediatric Hematology/Oncology, Fondazione IRCCS Policlinico San Matteo, Pavia, Italy; ^5^ Immunohaematology and Transfusion Medicine Service (SIMT), Fondazione IRCCS Policlinico San Matteo, Pavia, Italy

**Keywords:** cytotoxic T lymphocytes, haploidentical hematopoietic stem cell transplantation, adoptive cell therapy, pediatric acute leukemia, graft-versus-host-disease

## Abstract

Leuk-CTL-001 (EudraCT n. 2019-003362-41) is a Phase I/II clinical trial on the safety and preliminary efficacy of donor-derived anti-leukemia cytotoxic T lymphocytes (CTLs) for the prevention of leukemia relapse in children given haploidentical hematopoietic stem cell transplantation (HCT). The prognosis for children affected by acute leukemia and transplanted in an advanced disease stage, in the presence of measurable minimal residual disease (MRD) or with unfavorable cytogenetic abnormalities, is still poor and often less than 50%. Adoptive cell therapy based on the infusion of donor-derived CTLs able to recognize patients’ leukemia blasts (LB) is a promising approach to control leukemia relapse after allogeneic HCT. We previously described a procedure for *ex vivo* generating and expanding large numbers of donor-derived anti-leukemia CTL in compliance with Good Manufacturing Practice (GMP). The analysis of all batches of anti-leukemia CTLs produced so far documented that the majority of effector cells were CD3+/CD8+ cells, with a memory/terminal activated phenotype displaying efficient capacity to lyse patients’ LB and to secrete IFNγ and TNFα in response to leukemia cells. The Leuk-001 trial explores the safety of infusion of escalating doses of anti-leukemia CTLs in a cohort of high-risk relapse pediatric patients given haploidentical HCT for acute leukemia, starting within 60 days after transplantation. The safety is evaluated in terms of incidence of acute and chronic graft versus host disease (GVHD). The secondary objective is the evaluation of efficacy defined as cumulative incidence of relapse.

**Clinical Trial Registration:**

https://www.isrctn.com/, identifier ISRCTN13301166; https://clinicaltrials.gov/, NCT06865352.

## Introduction

The Italian age standardized incidence rate for acute lymphoblastic leukemia (ALL) in the 0–19 age population is 36.7 cases per million (95% CI 34.2-39.3) while, for acute myeloid leukemia (AML) it is 6.8 cases per million (95% CI 5.6-8.1). These incidence rates correspond to about 400 new diagnoses of pediatric ALL and 100 new diagnoses of pediatric AML per year in Italy. Even though, currently, more than 80% of children with ALL can be cured with conventional first line chemotherapy, 15-20% of children with ALL still present with disease relapse. The majority of relapsing patients are given an HCT after second-line chemotherapy, but only 30-70% can be cured by HCT (1). A subsequent relapse is the most frequent cause of treatment failure. Considering AML, the event-free survival probability for children treated with the AIEOP-AML 2002/01 protocol was 55% for the whole study population and 53% for high-risk patients (2) and leukemia relapse represented the most common cause of treatment failure. The probability of long-term survival for children relapsing after an allogeneic HCT is low: the vast majority die due to disease progression or for the complications of therapies.

Over the last four decades, allogeneic HCT from an HLA-matched donor, either related or unrelated, has been increasingly used to treat patients affected by various malignant or non-malignant disorders, including acute leukemia. However, only 25% of patients have an HLA-identical sibling and fewer than 60% of the remaining patients can be matched with suitable HLA-compatible, unrelated donors. In the absence of an HLA-matched donor, alternative donors, such as HLA-haploidentical relatives, are being increasingly used (3).

A study by The Acute Leukemia and Pediatric Working Parties of the European Society for Blood and Marrow Transplantation (EBMT) showed that the 5-year leukemia-free survival (LFS) for children with ALL, transplanted in complete remission was about 30%, indicating that haplo­HCT is a useful treatment for patients in morphological remission of disease (4). In particular, in the last years, T-cell receptor (TCR)αβ/CD19 cell depletion has emerged as an effective graft manipulation strategy for preventing GVHD in patients lacking HLA–matched donor and in need of an urgent transplant (5). Despite great improvements, leukemia relapse remains the most frequent cause of transplant failure, especially in high-risk patients, and the prognosis for children affected by acute leukemia and transplanted in an advanced disease stage, in the presence of measurable MRD or with unfavorable cytogenetic abnormalities is still poor, and often less that 50%. Further intensification of pre-transplant chemotherapy and conditioning regimen would increase the incidence of treatment-related toxicity and non-relapse mortality. Thus, in the last few years, clinical research has been directed towards the early identification of patients who cannot be cured by conventional treatment and who could benefit from the use of targeted therapy therapies (6–8).

Harnessing the cytotoxicity and targeting the ability of the cellular immune system could improve the efficacy of anticancer therapy. While the use of monoclonal antibodies is now well established in clinical practice, the development of cellular therapies against cancer has been slower, largely because of the complexity of this approach. Among the different forms of cellular immunotherapy, the adoptive transfer of T lymphocytes is the most promising to overcome leukemia resistance to chemotherapy. T cell therapy for solid and hematological tumors has proved to be effective in preventing or treating cancer growth in patients with different diseases such as melanoma, lymphoma, nasopharyngeal carcinoma (9, 10). Despite these results, cancer immunotherapy still has many limitations and obstacles. Most neoplasms can develop a range of immune escape strategies resulting in failure to appropriately present tumor antigens to immunocompetent cells. Other limitations of immunotherapy based on the infusion of T cells are the suboptimal persistence of transferred cells in the patient and the necessity to define the best tumor associated antigens (TAA) for cellular therapy.

In recent years, excellent results have been achieved in the control of relapsed/refractory ALL with the infusion of T lymphocytes genetically modified to express chimeric antigen receptors (CAR) targeting B cell-associated antigens (11–13). The increase in CAR-T cell efficacy, however, has been paralleled by the potential to induce severe adverse events, including cytokine release syndrome, B cell aplasia, and other severe on-target off-tumor toxicities (14). In addition, despite encouraging data on the treatment of B cell precursor ALL, major challenges remain to be overcome to safely apply CAR-T cell therapy to patients with other leukemia subtypes (T cell precursor ALL or AML) (15–17).

The use of autologous T cells may result in disparities in efficiency or yield of the final product, due to the patient’s prior treatment leading also to a manufacturing failure rate (18). This has led over the years to the development of allogeneic and/or ‘off-the-shelf’ CAR-Ts from healthy donors with the aim of providing a readily available therapeutic solution for patients who needed this therapy. CAR-T production using genome-editing as well as non-gene-editing technologies were evaluated. While these technologies have many advantages, they also have limitations due to associated safety risks, including inducing GVHD and rejection. At present, more extensive researches are required for the development of technologies that allow safe administration of allogeneic CAR-Ts while improving their persistence and their efficacy and maintaining a favorable safety profile (19).

The pivotal therapeutic role of immunity against acute leukemia has been revealed by the graft-versus-leukemia (GVL) effect observed following allogeneic HCT. Moreover, circulating leukemia-specific CTLs have been detected in patients with different forms of acute leukemia, and the presence of these specific T-cell responses in peripheral blood and bone marrow samples of leukemia patients has been associated with improved disease control and longer survival (20–23). This body of data suggests that allogeneic or naturally elicited leukemia reactive T cells could have an effect in preventing relapse and improving transplant outcome.

Unmanipulated donor lymphocyte infusion (DLI) is used after stem cell transplantation to treat and prevent relapse, to prevent infections and to establish full donor chimerism. However, an expected side effect of the presence of mature T cells is the potential occurrence of acute GVHD (24). Evidence has emerged that escalating DLI has achieved higher clinical response rates with lower GVHD occurrence (25). Optimization of DLI dose and schedule as well as strategies of donor T-cell manipulation may lead to the consistent ability to separate GVHD from GVL activity and improve the safety of DLI treatment. One way to manipulate donor lymphocytes to reduce GVHD is leukemia antigen stimulation, in order to increase antileukemia activity while reducing the number of alloreactive T cells by specific culture.

Somatic cell therapy with anti-leukemia CTLs may offer a new tool to prevent or treat relapse. The major advantage of immune-based therapies is the possibility to use highly selective immune effector cells directed against malignant cells, thus limiting treatment related toxicities.

## Pre-clinical background

Allogeneic or autologous anti-leukemia CTL directed against minor histocompatibility antigens or the BCR/ABL neoantigen (26–29), have been successfully employed to treat relapsed leukemia in adult patients representing proof of principle for the potential efficacy of this form of T cell therapy.

During the last decade, the proponent’s research unit has developed and optimized a procedure to generate donor-derived CTLs directed against pediatric acute LB, through the stimulation of peripheral blood mononuclear cells (PBMC) with IFN-dendritic cells (IFN-DC) pulsed with apoptotic LB as source of tumor antigens. CTLs generated *ex vivo* with this approach are likely to recognize a broader range of TAA, potentially reducing the risk of selecting variant leukemic subclones (30–32). Anti-leukemia CTLs include both effector and memory T-cells, suggesting the presence of lymphocytes able to exert, not only an immediate cytotoxic effector activity, but also to maintain long-term immune surveillance (33).

A major risk with the use of donor-derived anti-leukemia CTL is the subsequent development of GVHD. This risk is particularly relevant in the setting of haplo-HCT, where the donor and recipient are HLA partially-matched. In this regard it has been documented that although some anti-leukemia CTL lines showed sizeable cytotoxicity against patients’ derived PHA-blasts, the vast majority displayed lower levels of alloreactivity compared with that observed against LB, especially at the lowest E:T (31, 32).

Any successful cell therapy approach strongly depends on the possibility to *in vitro* generate a product with a high level of standardization in compliance with GMP. Anti-leukemia CTL are Advanced Therapy Medical products (ATMP) and GMP guidelines ensure their quality and safety in terms of sterility, purity and potency for *in vivo* use. Since 2016, after optimizing protocols for obtaining highly specific anti-leukemia CTL, ATMP have been prepared in the Fondazione IRCCS Policlinico San Matteo’s GMP facility “Cell Factory”.

In a recent paper (34), we reported data obtained in 51 batches of ATMP documenting that biological QC, including cell viability, identity, phenotype and potency were in compliance with the defined cut offs. We also deeply evaluated the phenotypic and functional features of ATMP batches using biological assays, other than those necessary for batch release. By comparing the ability of each ATMP to lyse LB in the cytotoxicity assay and to secrete IFN-γ and TNF-α in response to LB, we documented that the majority of ATMPs displayed sizeable levels of cytotoxic activity against LB and high percentages of cytokine-secreting cells. No significant differences were documented in the potency of ATMP obtained after 1^st^ and the 2^nd^ round of rapid expansion. In few ATMP derived from different donors, unable to mount sizable levels of cytotoxic activity against patients LB, high percentages of IFN-γ and/or TNF-α-secreting cells were documented. These data suggested that anti-leukemia CTL are able to mediate anti-leukemia activity by different mechanisms and that the secretion of cytokines with anti-tumor activity can make up for low levels of direct lytic activity (34).

ATMP were also characterized for surface antigens of terminal differentiation and exhaustion, which can be an indirect sign of impaired function or persistence. The analysis of all batches of ATMP produced so far documented that the majority of cells were CD3+/CD8+ cells, with a memory/terminal activated phenotype including also measurable percentages of T central memory (TCM). Anti-leukemia CTL appear more highly differentiated than exhausted in that we also documented a low percentages of the PD1+/TIM3 population, usually associated with an exhausted phenotype. In donor/recipient pairs in which more than one batch of ATMP was produced, biological QC and additional biological assays documented that they were homogeneous in terms of surface antigens and potency (34).

Altogether these results demonstrated that the methodological approach we have optimized protocol is highly reproducible and allows the generation of large numbers of immunologically safe and functional anti-leukemia CTL with a high level of standardization. Based on their features, anti-leukemia CTLs could be a safe and efficient somatic cell therapy to prevent/treat leukemia relapse in children given haplo-HCT for high-risk acute leukemia.

## Methods and analysis

### Trial design

**Figure 1:**
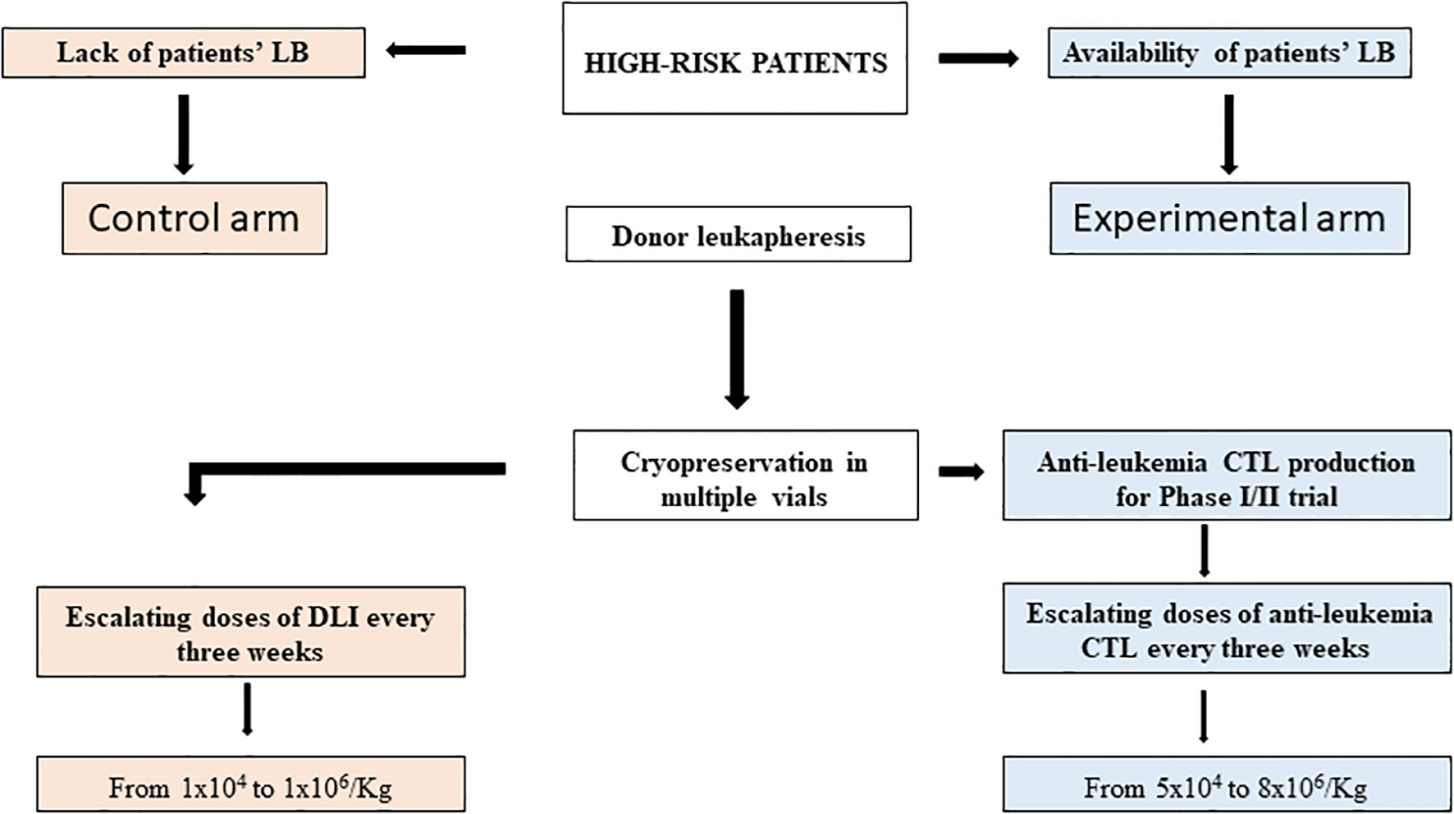
Trial design flow chart. The study population includes high-risk ALL or AML pediatric patients candidate to haplo-HCT and with an expected risk of leukemia relapse after transplantation ≥ 50%. The donor, once screened and deemed eligible, will undergo leukapheresis to obtain PBMC cryopreserved in multiple vials. Depending on the availability of cryopreserved LB patients will be assigned to the treatment with anti-leukemia CTLs (experimental arm), otherwise they will proceed to standard treatment with DLI (control arm).

Leuk-001 is a Phase I/II, monocentric, open label, non-randomized, prospective clinical trial of donor-derived anti-leukemia CTLs in pediatric haplo-HCT recipients transplanted for ALL or AML and with a risk of leukemia relapse after haplo-HCT ≥ 50%. The outcome of treated patients will be compared with that of pediatric recipients of haplo-HCT with the same disease characteristics and prognosis, who received infusions of unmanipulated DLI. Patients will be assigned to the treatment with anti-leukemia CTLs (experimental arm) or to standard treatment with DLI (control arm) based on the availability of cryopreserved viable LB collected at the diagnosis or at relapse, necessary for the production of anti-leukemia CTLs (**Figure 1**). Therefore, this will be the only difference between the study population and the control population.

### Study objectives

The primary objective will be safety, measured as the incidence of acute GVHD after treatment. Acute GVHD will be diagnosed and graded according to the NIH criteria. Grade II-IV acute GVHD will be expressed as cumulative incidence considering disease relapse and death in remission without GVHD as competing events. The key secondary objective will be preliminary efficacy, measured as the incidence of relapse, (REL) defined as the time from HCT to the date of disease relapse, will be calculated at 3, 6, 9, 12–18 and 24 months after HCT and expressed as cumulative incidence considering death in remission as competing event.

### Patients’ selection

Fifteen pediatric subjects will be enrolled in this study after obtaining informed consent. The study population will comprise any infant (1 month- 24 months), child (2–11 years) and teenager (12–18 years) affected by high-risk ALL or AML, candidate to an haplo-HCT and with an expected risk of leukemia relapse after transplantation ≥ 50% according to the available literature data. In **Tables 1**, **2** patients’ inclusion and exclusion criteria are reported, respectively. Eligible donors are HLA haploidentical relatives, including but not limited to biological parents, siblings, or half-siblings. Matching will be determined by class I and class II DNA typing. In **Table 3**, donor inclusion and exclusion criteria are reported.

**Table 1:** Inclusion criteria for ALL and AML patients’ enrolment.

ALL patients’ inclusion criteria	AML patients’ inclusion criteria
Age ≥ 1 month and ≤ 18 years	Age ≥ 1 month and≤ 18 years
Life expectancy > 12 weeks	Life expectancy > 12 weeks
ALL in first morphological remission but with a positive MRD≥ 1 x 10–^3^ before HCT	AML in first morphological remission and with a flow cytometry MRD at the end of induction therapy ≥ 0.1%;
ALL in second morphological remission after a high-risk relapse (patients belonging to the S3-S4 BFM risk group), independently of the level of MRD	AML in first morphological remission and with high-risk disease according to the presence of unfavorable cytogenetic or molecular aberrations
ALL in second morphological remission with any MRD positivity before HCT	AML in first morphological remission after a primary induction failure
ALL in third or subsequent morphological remission, independently of the level of MRD	AML in second morphological remission
ALL patients not in morphological remission at time of HSCT	AML in third or subsequent morphological remission
Pre-HSCT Lansky/Karnofsky score ≥ 40%.	Pre-HSCT Lansky/Karnofsky score ≥ 40%.
HIV negativity	HIV negativity

**Table 2:** Exclusion criteria for patients’ enrolment.

Patients’ exclusion criteria
Ongoing active acute GVHD or chronic GVHD due to a previous allograft
Presence of clinically active infectious disease (including positive HIV serology or viral RNA)
Severe cardiovascular disease (arrhythmias requiring chronic treatment, congestive heart failure or left ventricular ejection fraction <40%)
Liver dysfunction (AST/ALT ≥ 3 times institutional upper limit normal value –ULN- or bilirubin > 3 times ULN)
Renal dysfunction: serum creatinine > 1.5 times ULN or calculated creatinine clearance < 60 ml/min/1.73 m^2^
End stage irreversible multi-system organ failure.
Other active malignancy.
Pregnant or breast feeding female patient
Lack of parents’/guardian’s written informed consent for children who are minors or lack of written informed consent for patients aged 18 y

**Table 3:** Parameters for batch release.

Parameter	Methodology	Cutoff value
Sterility	Automated culture method	negative
Bacterial endotoxin content	LAL test	<0.5 EU/ml
Mycoplasma content	RT-PCR	absent
Viability	Trypan blue staining	>80%
Genotype identity	Molecular analysis	- molecular identity between ATMP, starting material and donor - absence of foreign genetic material
Phenotype	Flow cytometry analysis	T-CD3+ ≥ 75%, CD3-neg/CD56 + 0-25%, CD19+/CD20+ CD14+ ≤ 5%
Potency	Cytotoxicity by ^51^Cr assay ^¶^	% specific lysis >45%

Microbiologic controls of ATMP were performed under aseptic conditions, according to European Pharmacopoeia (Eu.Ph.) guidelines. ¶ Potency was evaluated by CD3-redirected assay against P815 cell line.

### Anti-leukemia CTLs production

The production of anti-leukemia CTLs is carried out in compliance with current European GMP regulations. The active substance consists of donor T lymphocytes with cytotoxic capacity directed against LB of the patient for whom the cell therapy medicinal product is intended. Starting material consists of mononuclear cells obtained by HCT donor. The donor once screened and deemed eligible, before mobilization for hematopoietic stem cell collection, will undergo leukapheresis, following the established regulatory guidelines for monitoring and institutional standard procedures for collection. PBMC will be isolated by density gradient centrifugation and cryopreserved. Patients’ LB are collected and cryopreserved at the time of leukemia diagnosis/recurrence. The methodological approach for the production of anti-leukemia CTL was optimized over time and consists in three different phases: priming, leukemia-specific stimulation and rapid antigen independent expansion. Priming is based on the use of donor DC, derived from CD14+ cells cultured four days in the presence of rGM-CSF and IFN-α2b, pulsed with irradiated (200Gy) apoptotic patients’ LB as the source of leukemia associated antigens, as previously described (31) in medium supplemented with appropriate concentration of IL-7, IL-12, IL-15 and IFNα 2b (34).

**Figure 2:**
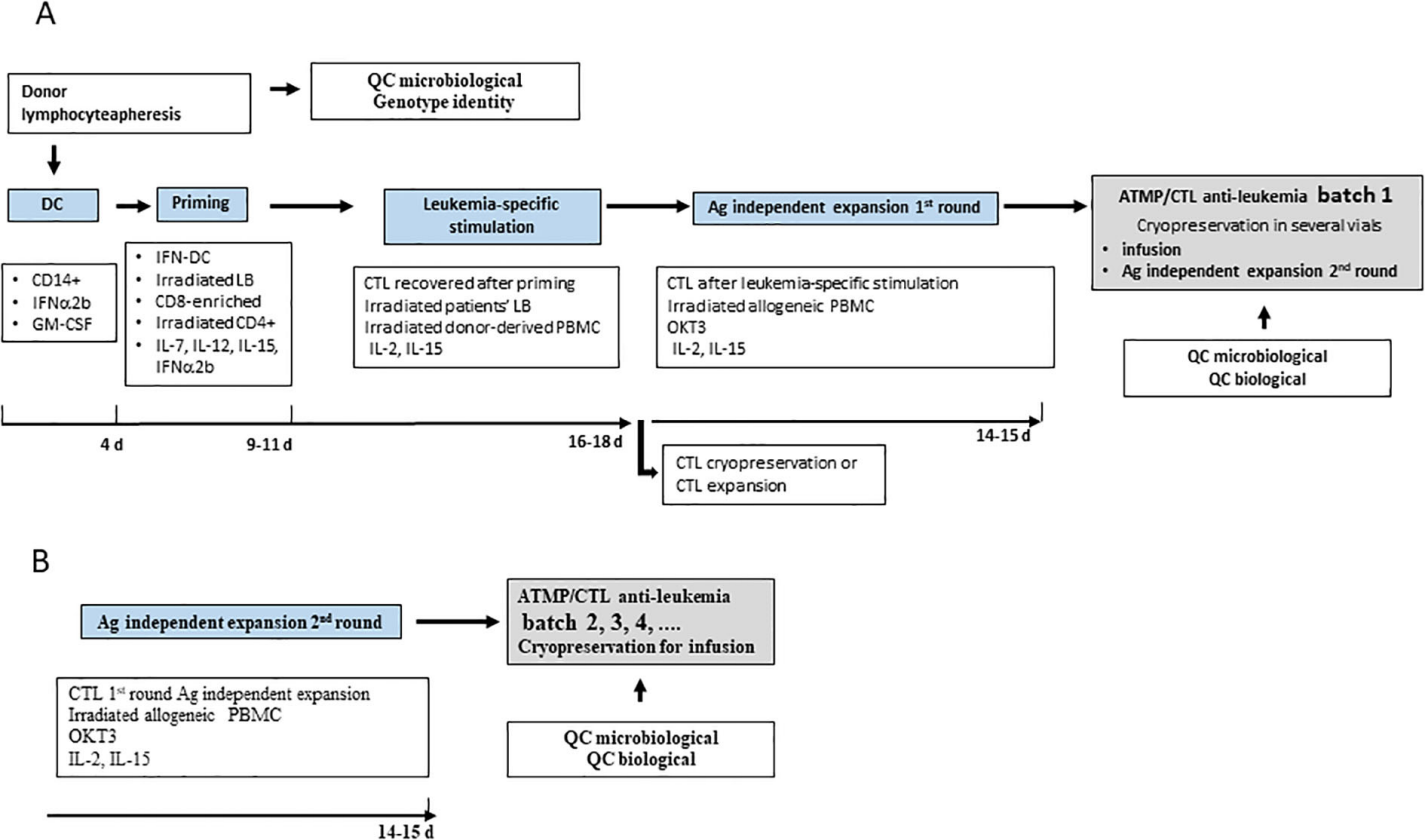
Protocol of production of anti-leukemia CTL. **(A)** After priming and leukemia-specific stimulation, CTL underwent the 1**^st^** round of antigen independent expansion. Anti-leukemia CTL recovered after the 1^st^ round of rapid expansion were cryopreserved in several vials. Some vials represent the batch 1 of ATMP and can be infused to patients or alternately undergo a 2**^nd^** round of rapid expansion **(B)**, to produce the next batches of ATMP. Batch 1 and subsequent ones were subjected to microbiological and biological quality controls for the release of ATMP.

As shown in **Figure 2**, after priming and leukemia-specific stimulation, CTLs undergo the 1^st^ round of antigen independent expansion. After leukemia-specific stimulation, CTLs are recovered and can be cryopreserved or undergo the first round of rapid expansion. Anti-leukemia CTLs recovered after the 1^st^ round of rapid expansion are cryopreserved in vials. Batch 1 of the ATMP can be administered to patients or, if necessary, undergo a subsequent round of rapid expansion for the production of further batches of ATMP. Following this protocol, virtually billions of CTLs can be obtained for each patient. The time necessary to expand CTL batch 1 is just over a month, while subsequent batches are produced in about two weeks. At the end of the culture, CTLs mostly contain CD3+/CD8+ T lymphocytes, and a low percentage of CD3+/CD4+ T lymphocytes. ATMP batches are subjected to microbiological and biological quality controls (QC) before product release. Microbiological QC include testing for sterility, endotoxin by LAL assay and Mycoplasma by RT-PCR. Biological QC include: i) count of viable cells before and after cryopreservation; ii) immune phenotype characterization; iii) genotypic identity; iv) potency by means of CD3-redirected cytotoxicity test (**Table 3**). After confirming the stability of ATMP up to eight years after cryopreservation, biological CQ that included evaluation of phenotype and potency are performed on cryopreserved ATMPs as they represent the product that will be infused.

### Study procedures

Anti-leukemia CTLs will be administered at progressively increasing doses every 3 weeks according to the following schedule: 5x10^4^/kg; 1x10^5^/kg; 5x10^5^/kg; 1x10^6^/kg; 2x10^6^/kg; 4x10^6^/kg; 8x10^6^/kg and 8x10^6^/kg. Treatment will start within 60 days after transplantation, and it will continue for the next 6–8 months, depending on the frequency of CTL infusions. In the absence of complications, the minimum number of infusions is eight. Considering the high risk of recurrence, based on the clinician’s judgement, it will be possible to administer subsequent monthly CTL infusions, at the same dose used for the last administration, until month +12 from HCT.

Anti-viral prophylaxis will be administered per standard site procedures. During the treatment, the administration of corticosteroids should be avoided, unless necessary, and in this case it will be recorded.

In case of development of grade I acute GVHD, subsequent CTL administrations will be delayed by 1 week and CTLs will be given at the immediately lower dose than that which preceded the occurrence of GVHD. Subsequently, the treatment will continue with the same schedule. In case of development of acute GVHD of grade ≥ II, treatment will be stopped until complete resolution of GVHD. Subsequently, based on the clinician’s judgement, anti-leukemia CTLs may be resumed, starting from the immediately lower dose than that which preceded the occurrence of GVHD, every 3 weeks with the same schedule. Subjects who develop grade I or II skin GVHD (up to Stage 3 skin GVHD without any gut or liver involvement) will be treated with topical steroids and/or other standard of care (SOC) therapies. Subjects who experience Grade III skin and/or Grade II- III non-cutaneous GVHD will be treated with standard therapy. Chronic GVHD will be treated according to SOC. In **Table 4** the stopping rules are reported.

**Table 4:** Stopping rules.

Stopping rules
Development of grade III-IV acute GVHD in 2 out of the first 4 patients treated.
Development of any severe adverse event (AE) in 2 out of the first 4 patients treated.
Development of any serious adverse event (SAE) in 2 of the 4 patients of each subsequent group of 4 patients.
Incidence of grade II-IV acute GVHD ≥ 40% in the enrolled patient population.
Incidence of chronic GVHD (mild, moderate or severe) ≥ 40 in the enrolled patient population.

A screening evaluation that includes clinical and laboratory assessment will be performed at enrollment, prior to each CTL infusion, and at day 30, 90, 180, 360, 540 and 720 from HCT (if they do not coincide with CTL infusions). Prior to each CTL infusion, chimerism analysis and immunological follow up on peripheral blood will be performed. The immunological follow up will include evaluation of the percentages of circulating T, Treg, B and NK subsets, interferon-gamma secreting cells in response to LB stimulation *in vitro* on peripheral blood. MRD evaluation on bone marrow will be performed at baseline, at +90, +180 and +360 days after Haplo-HCT.

### Toxicity evaluation

Safety assessments will consist of monitoring protocol-defined endpoints, such as acute GVHD, measurement of protocol-specified hematology, clinical chemistry variables, vital signs and other protocol-specified tests that are deemed critical to the safety evaluation of the ATMP. All adverse events (AEs) and serious adverse events (SAEs) will be recorded. AEs will be collected for 30 days following the final infusion of anti-leukemia CTLs. All SAEs will be collected until 90 days following the last anti-leukemia CTL infusion. After this period, investigators will report SAEs considered related to the study treatment.

### Sample size and data analysis

The sample size is calculated based on the primary safety endpoint, using the single stage method for phase II studies proposed by Fleming (35). This method has been primarily designed to provide sufficient clinical experience to support the design of later-stage clinical development such as phase III studies. We assumed a maximum proportion P0 of patients developing GVHD, for patients receiving the standard treatment (DLI), of 40% based on our previous experience, and on data reported in the literature (H0). We consider 10% as the maximum proportion P1 of patients developing GVHD for the study treatment (CTLs) to consider the study treatment for preliminary success (H1). A sample size of 15 patients (including a dropout of 1 patient) will be able to reject H0 with a power of 80% and a type I error of 5%. If the number of responses at the end of the study is ≤ 2, H0 will be rejected; if the number of responses is > 2, H1 will be rejected. Quantitative variables will be reported as the median value and range, while categorical variables will be expressed as absolute numbers and percentage. The demographic and clinical characteristics of patients and controls will be compared using the Chi-square test or Fisher’s exact test for categorical variables, while the Mann-Whitney rank sum test or the Student’s t-test will be used for continuous variables as appropriate. Overall survival (OS) and event-free survival (EFS) will be calculated according to the Kaplan-Meier method, while the risk of acute and chronic GVHD, relapse and death in remission, defined as non-relapse mortality (NRM) will be calculated as cumulative incidences (CI) in order to adjust the analysis for competing risks. Comparisons between different OS and EFS probabilities will be performed using the Log-Rank test, while the Gray’s test will be used to assess, in univariable analyses, differences between cumulative incidences. All results will be expressed as probabilities (%) or cumulative incidences (%) and 95% confidence interval (95% CI), at 3, 6, 9, 12, 18 and 24 months from HCT. P values < 0.05 will be considered statistically significant. Statistical analysis will be performed using NCSS [NCSS 10 Statistical Software (2015). NCSS, LLC. Kaysville, Utah, ncss.com/software/ncss.] and MP/15 (StataCorp LP, 4905 Lakeway Drive, College Station, TX 77845 USA (http://www.stata.com).

## Discussion

This study explores the use of escalating doses of donor-derived anti-leukemia CTL to prevent leukemia relapse in high-risk pediatric patients affected by acute leukemia and given haplo-HCT. The primary objective is the incidence of acute GVHD compared with that observed in a control cohort of patients with identical characteristics, treated with infusion of unmanipulated DLI. Haplo-HCT based on selective depletion of TCRα/β+ and CD19+ cells has been increasingly used in pediatric patients affected by high-risk acute leukemia. By this kind of graft manipulation, T cells expressing the αβ chains of the T cell receptor, which are responsible for the development of GVHD, are removed with a high depletion efficiency (36). However, the almost complete elimination of T cells on the graft increases the risk of infections and relapse. Infusion of donor-derived mature T cells represents a way to overcome these complications. Over the years, several strategies of donor-T cell manipulation prior transfer in patients have been proposed to improve the outcome of patients without the emergence of acute and chronic GVHD that represent the most common complications after DLI. Results of clinical trials documented that the transfer of selective populations of mature T cells may improve the recovery of pathogen-specific immunity but there are no significant data regarding the incidence of relapse (37, 38).

Anti-leukemia CTLs obtained by stimulation with patients’ LB are specific for each individual blast signature, and, due to their physiological recognition and effector mechanism through their natural T cell receptor, exert leukemia-specific killing with less severe adverse reactions than CAR-T cells. In addition, their potential to recognize multiple leukemia-associated antigens present on the blast surface should make them less susceptible to immune evasion strategies developed by leukemic cells. Additionally, the risk of GVHD should be reduced by the culture procedure, which decreases the number of alloreactive T cells. For these reasons, the use of these T cells after HCT in a highly personalized approach may be a safer and more effective option than unmanipulated DLI to prevent leukemia relapse after HCT. To support this hypothesis, in a pioneer study performed in patients treated on a compassionate basis, we documented that the infusion of large numbers of anti-leukemia CTL is safe, as no patient experienced acute or chronic GVHD or severe immune adverse reactions, and may contribute to the restoration of CR in a proportion of patients relapsing after the allograft. In case of overt clinical relapse, prior reduction of the tumor burden may facilitate the anti-leukemia effect displayed by CTLs (39).

Overall, the results of this clinical study may open the way to a new approach, different from CAR-T cell therapy, for the management of high-risk patients given haplo-HCT. Overall, analysis of the features of ATMP produced so far documented that there is a certain degree of homogeneity in terms of potency and surface markers among ATMP batches in different donors and among batches produced from the same donor. Batches from different donors could show variability in lytic capacity always within the acceptable release criteria range. The evaluation of immunological properties of the infused anti-leukemia CTL and clinical follow up of treated patients could allow a better understanding of how the functional and phenotypical CTL characteristics may affect patients’ outcome.

## Data Availability

The original contributions presented in the study are included in the article/supplementary material. Further inquiries can be directed to the corresponding author.
